# The optimal regimen of oral tranexamic acid administration for primary total knee/hip replacement: a meta-analysis and narrative review of a randomized controlled trial

**DOI:** 10.1186/s13018-020-01983-1

**Published:** 2020-10-06

**Authors:** Wei Ye, Yafang Liu, Wei Feng Liu, Xiao Long Li, Jianshu Shao

**Affiliations:** 1 Department of Orthopedics Medicine, Wujin Hospital Affiliated with Jiangsu University, Changzhou, 213000 China; 2 Department of Respiratory Medicine, The Wujin Clinical college of Xuzhou Medical University, Changzhou, 213000 China

**Keywords:** Oral, Multiple dose, Tranexamic acid, Total knee/hip arthroplasty

## Abstract

**Background:**

Oral tranexamic acid (TXA) has been demonstrated to reduce the blood loss in primary total knee and hip arthroplasty, but the optimal regimen of oral TXA administration is still unknown. This study aimed to find the best number of administrations of oral TXA for primary total knee and hip arthroplasty.

**Methods:**

The PubMed, Embase, and Cochrane Library databases were searched for relevant studies published before March 20, 2020. Studies clearly reporting a comparison of multiple administrations of oral TXA for total hip/knee replacement were included, and the total blood loss (TBL), intraoperative blood loss (IBL), decline in hemoglobin (DHB), deep vein thrombosis (DVT), intramuscular venous thrombosis (IVT), length of hospital stay (LOS), and transfusion rate were evaluated. The weighted mean differences and relative risks were calculated using a fixed effects or random effects model.

**Results:**

Nine studies involving 1678 patients were included in this meta-analysis (TXA 1363 (one administration, 201; two administrations, 496; three administrations, 215; four administrations, 336; five administrations, 115); placebo 315); the results show that compared with placebo groups, oral TXA could significantly reduce the TBL, IBL, DHB, LOS, and transfusion rate. In addition, the incidences of IVT and DVT were similar between the TXA and placebo groups. Moreover, two administrations of oral TXA significantly reduced the TBL and DHB compared with one administration, three administrations of oral TXA were better than two administrations, and four administrations of oral TXA were better than three administrations.

**Conclusion:**

Our results suggested that oral TXA could significantly reduce the blood loss and the length of hospital stay but could not increase the incidence of DVT and IVT for total joint replacement patients; additionally, the effectiveness of oral TXA administration increased as the number of administrations increased.

## Introduction

Total joint arthroplasty (TKA/THA) is a reliable surgical method for patients who suffer from moderate-to-severe degenerative knee or hip joint diseases and osteoarthritis pain, but controlling perioperative bleeding is a major problem for orthopedic surgeons. Many previous studies have reported that the estimated total blood loss was between 800 ml and 1800 ml in total joint replacement patients [1, 2]. To overcome this problem, several methods have been used to reduce perioperative blood loss, such as tourniquet use, blood transfusions, administration of iron supplements, and the application of anti-fibrinolytic drugs [3–6]. Transformations have various limitations, such as prolonging the patient’s rehabilitation time and extending the length of hospital stay. In addition, transfusions incur a considerable cost and are associated with the risk of side effects, infectious diseases, inhibition of the immune system, and so on [7–9]. Tranexamic acid (TXA) is a synthetic agent that exerts its antifibrinolytic effects by inhibiting plasminogen. TXA inhibits plasminogen activation by binding plasmin to fibrin, which leads to clot stabilization and reduces blood loss. In addition, many previous publications have confirmed that TXA is a simple, inexpensive, and effective drug for reducing perioperative blood loss in total knee arthroplasty regardless of intravenous, intra-articular injection, or oral administration [10–14]. However, there is still no consensus about which route is the best for administering TXA to total joint arthroplasty patients, and in recent years, some studies have recommended multiple administrations of oral TXA. However, there is still no consensus regarding the optimal regimen of oral tranexamic acid administration; therefore, we conducted a study to determine the most effective regimen of oral TXA for primary total knee and hip arthroplasty patients.

## Materials and methods

### Search strategy

Pubmed, Cochrane library, and Embase databases were searched independently by two investigators to retrieve relevant studies published before March 20, 2020. The search criteria “total knee/hip arthroplasty,” “TKA/THA,” “total joint replacement,” ”TKR/THR,” “tranexamic acid,” “TXA”, ”oral,” and ”multiple dose” were used in key words for search. All studies selected were reviewed independently by the authors and examined for broadening the potential studies through the “related articles” function. Thus, the reference lists of the included articles were also manually checked to find relevant studies that were not found during the database searches.

#### Inclusion criteria

(1) The adult patients with knee or hip joint degenerative disease and received primary TKA/THA, (2) TXA oral administration include different dose groups, (3) examination includes anyone among total blood loss (TBL), intraoperative blood loss (IBL), the decline in hemoglobin (DHB), deep vein thrombosis (DVT), intramuscular venous thrombosis (IVT), the length of hospital stay (LOS), and the transfusion rate.

#### Exclusion criteria

(1) Total joint replacement is not for joint degenerative diseases such as trauma, tumors, or bilateral joint replacement; (2) study only report one oral group or case report; and (3) animal or laboratory study.

### Data extraction

Each article’s variables and outcomes of interest and assessment of the methodological quality were reviewed independently by two readers. If there was a difference of opinion, the problems were resolved through discussion and consensus. The methodological quality of the trials was assessed through the Cochrane Handbook for Systematic Reviews of Interventions 5.1.

### Statistical analysis

The statistical analysis was performed using Review Manager 5.1 for Windows System (Cochrane Collaboration, Nordic Cochrane Centre, Copenhagen, Denmark). Categorical dichotomous variables were analyzed with relative risks (RRs), continuous variables were assessed with the weighted mean difference, and *P* < 0.05 was considered statically significant; the 95% confidence intervals (CIs) were reported. Heterogeneity was considered significant if the *P* value was less than 0.1. The value of *I*^2^ statistics was used to assess the degree of heterogeneity (*I*^2^ < 25%, no heterogeneity; *I*^2^ = 25–50%, moderate heterogeneity; *I*^2^ > 50%, large or extreme heterogeneity); if *I*^2^ > 50%, a fixed-effects model was used. The presence of publication bias was assessed by a visual inspection of a funnel plot and the Begg and Egger tests (with *P* < 0.05 considered statistically significant).

## Results

### Literature search

**Fig. 1:**
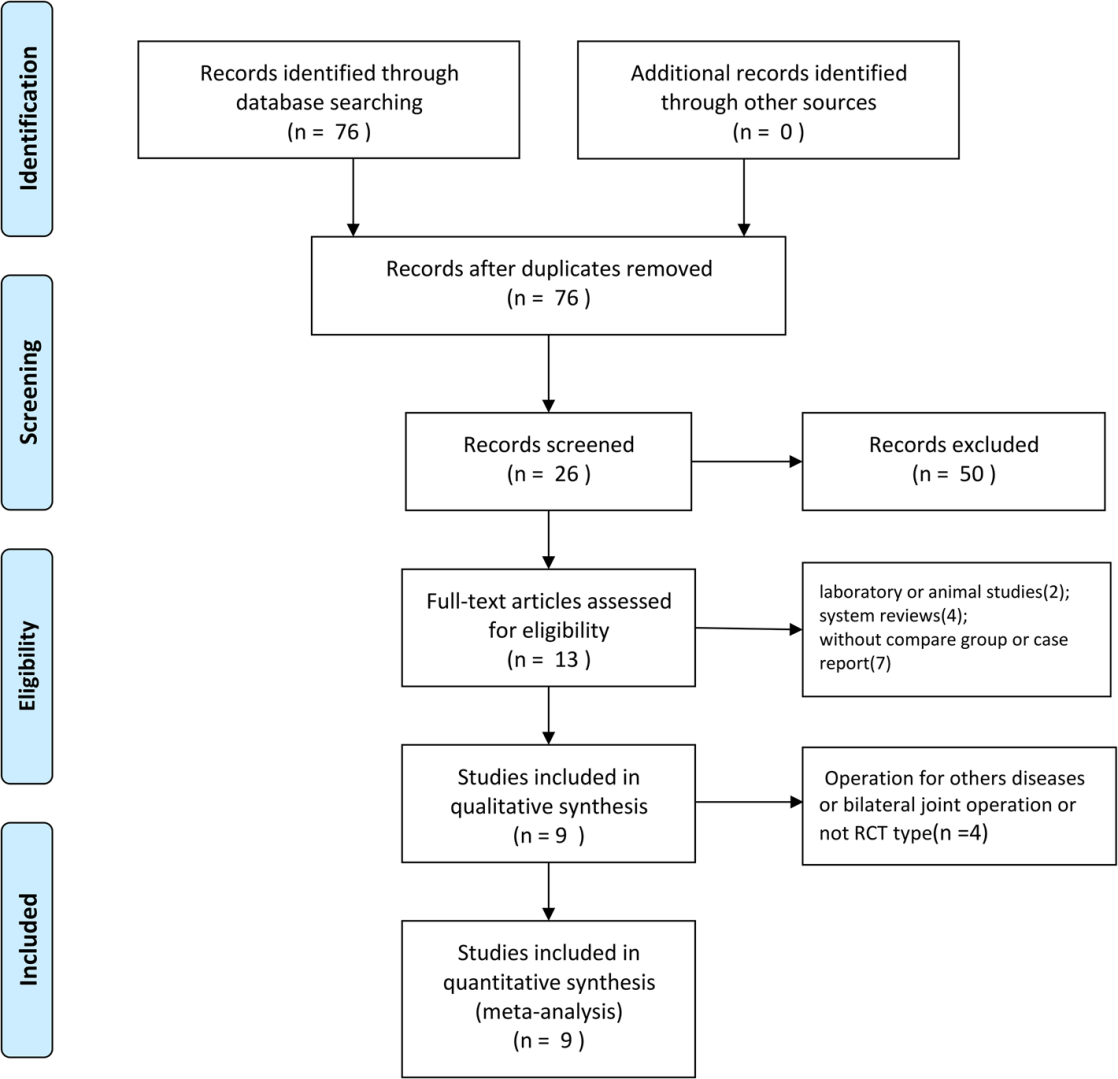
Search strategy flow diagram

**Fig. 2:**
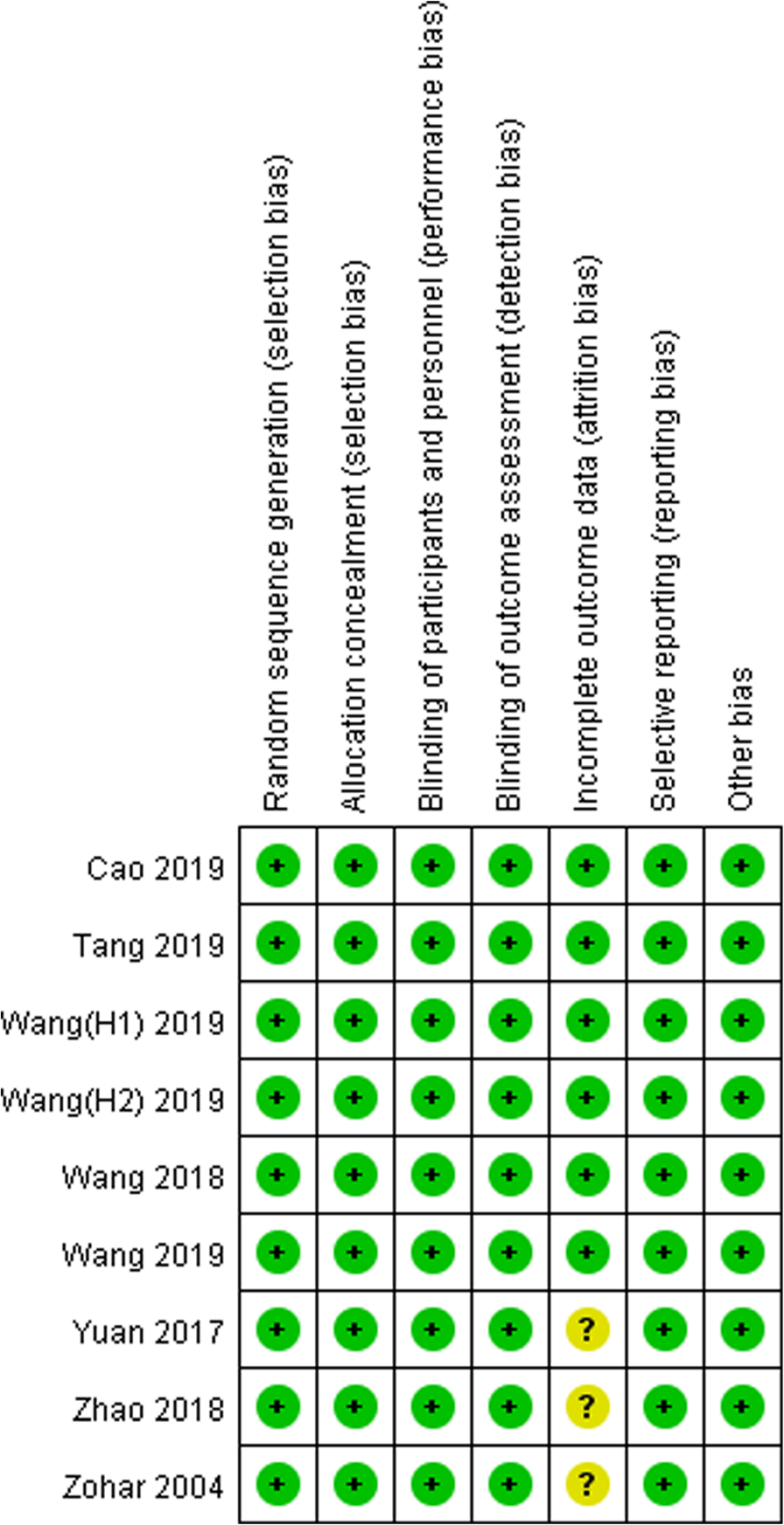
Summarizes the methodological quality of the selected studies

The initial literature search retrieved 76 relevant articles (duplicates were discarded). After a careful screening of the titles, 50 articles were excluded for not investigating the topic of interest. After reviewing the abstracts, 13 articles were excluded (2 laboratory or animal studies, 4 not RCT type, 7 without compare group or case report). After reading full articles, 4 studies were excluded for the operation for other diseases or bilateral joint operation or not RCT type. Therefore, leaving 9 studies that matched the selection criteria and were suitable for meta-analysis (Fig. 1). A total of 1678 (TXA 1363 (once, 201; twice, 496; thrice, 215; quartic, 336; quintic, 115); placebo 315) patients were enrolled in the studies, and the information of the included studies is summarized in Table 1 [15–23]. They are all RCT studies and the methodological bias of this study was low (Fig. 2).

**Table 1:** The information of included studies

Study	Country	H/K	Placebo	TXA	TXA administration
Wang et al. 2018 [18]	China	K	0	Once:50 Twice:50 Thrice:50 Quartic:50	Once: 2 g at 2 h pre-operatively Twice: 2 g at 2 h pre-operatively and 1 g at 3 h post-operatively Thrice: 2 g at 2 h pre-operatively and 1 g at 3 and 9 h post-operatively Quartic: 2 g at 2 h pre-operatively and 1 g at 3, 9, and 15 h post-operatively
Wang et al. (H1) 2019 [19]	China	H	0	Once:50 Twice:50 Thrice:50 Quartic:50	Once: 2 g at 2 h pre-operatively Twice: 2 g at 2 h pre-operatively and 1 g at 3 h post-operatively Thrice: 2 g at 2 h pre-operatively and 1 g at 3 and 9 h post-operatively Quartic:2 g at 2 h pre-operatively and 1 g at 3, 9, and 15 h post-operatively
Tang et al. 2019 [16]	China	K	0	Once:50 Twice:50 Quartic:51	Once: 2 g at 2 h pre-operatively Twice: 2 g at 2 h pre-operatively and 4 h post-operatively Quartic: 2 g at 2 h pre-operatively and 4, 10, and 16 h post-operatively
Wang et al. (H2) 2019 [20]	China	H	60	Twice:60 Thrice:60 Quartic:60 Quintic:60	Twice: 2 g at 2 h pre-operatively and 1 g at 3 h post-operatively Thrice: 2 g at 2 h pre-operatively and 1 g at 3 and 7 h post-operatively Quartic: 2 g at 2 h pre-operatively and 1 g at 3, 7, and 11 h post-operatively Quintic: 2 g at 2 h pre-operatively and 1 g at 3, 7, 11, and 15 h post-operatively
Wang et al. 2019 [17]	China	K	55	Twice:55 Thrice:55 Quartic:55 Quintic:55	Twice: 2 g at 2 h pre-operatively and 1 g at 3 h post-operatively Thrice: 2 g at 2 h pre-operatively and 1 g at 3 and 7 h post-operatively Quartic: 2 g at 2 h pre-operatively and 1 g at 3, 7, and 11 h post-operatively Quintic: 2 g at 2 h pre-operatively and 1 g at 3, 7, 11, and 15 h post-operatively
Cao et al. 2019 [15]	China	H	0	Once:51 Twice:51 Quartic:50	Once: 2 g at 2 h pre-operatively Twice: 2 g at 2 h pre-operatively and 4 h post-operatively Quartic: 2 g at 2 h pre-operatively and 4, 10, and 16 h post-operatively
Yuan et al. 2017 [21]	China	K	140	Twice:140	Twice: 2 mg/kg at 2 h pre-operatively and 12 h post-operatively
Zhao et al. 2018 [22]	China	H	40	Twice:40	Twice: 2 mg/kg at 2 h pre-operatively and 3 h post-operatively
Zohar et al. 2004 [23]	China	K	20	Quartic:20	Quartic: 1 g at 1 h pre-operatively and 6, 12, and 18 h post-operatively

### Main analysis

**Fig. 3:**
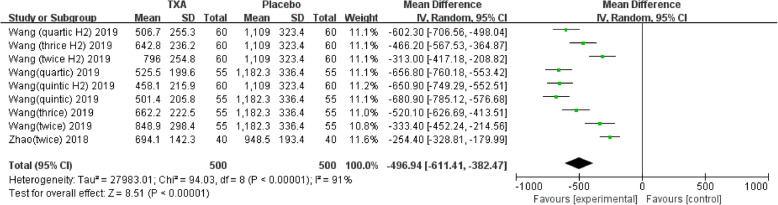
Forest plot showing the weighted mean difference in total blood loss (TBL) between oral and placebo groups

**Fig. 4:**
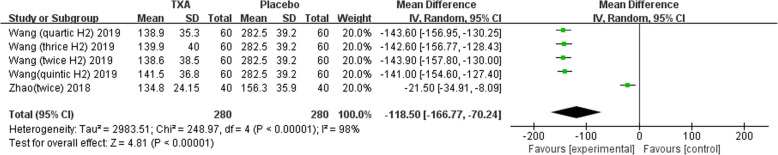
Forest plot showing the weighted mean difference in intraoperative blood loss (IBL) between oral and placebo groups

**Fig. 5:**
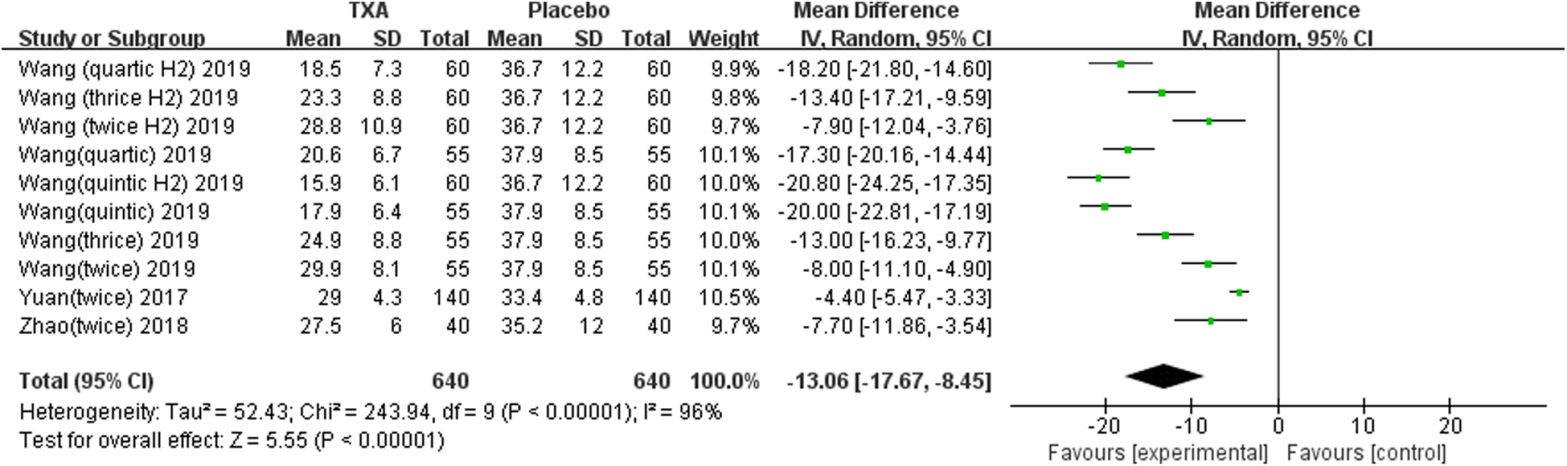
Forest plot showing the weighted mean difference in the decline of hemoglobin (DHB) between oral and placebo groups

**Fig. 6:**
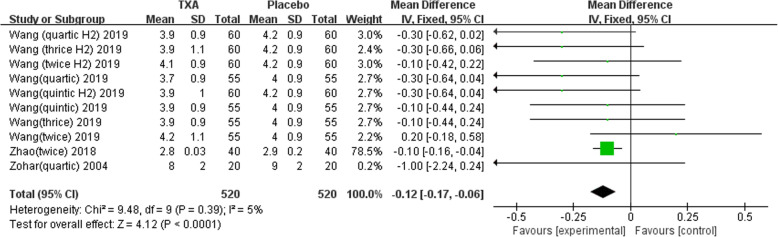
Forest plot showing the weighted mean difference in the length of hospital stay (LOS) between oral and placebo groups

**Fig. 7:**
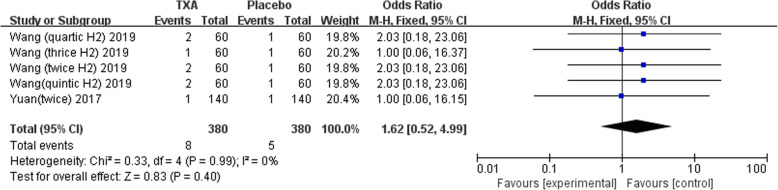
Forest plot showing the OR difference in DVT rate between oral and placebo group

**Fig. 8:**
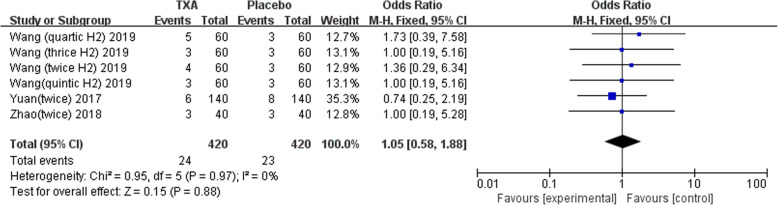
Forest plot showing the OR difference IVT rate between oral and placebo group

**Fig. 9:**
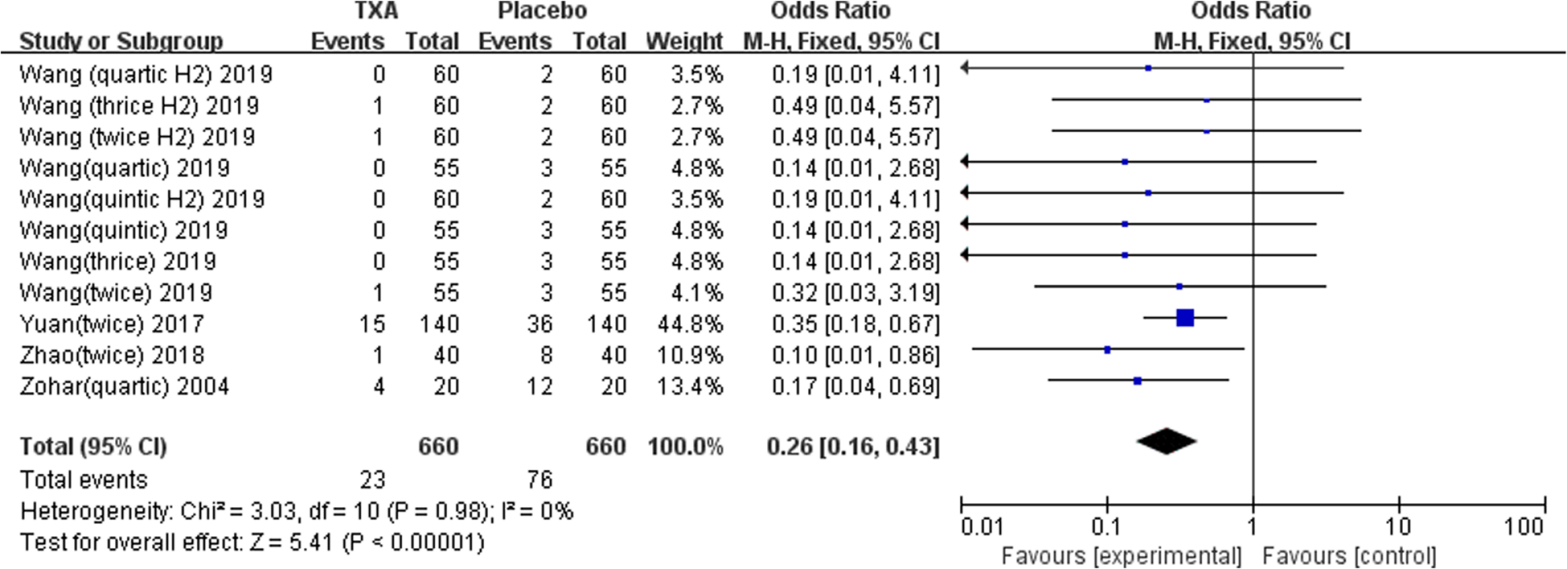
Forest plot showing the OR difference in transfusion rate between oral and placebo groups

**Fig. 10:**
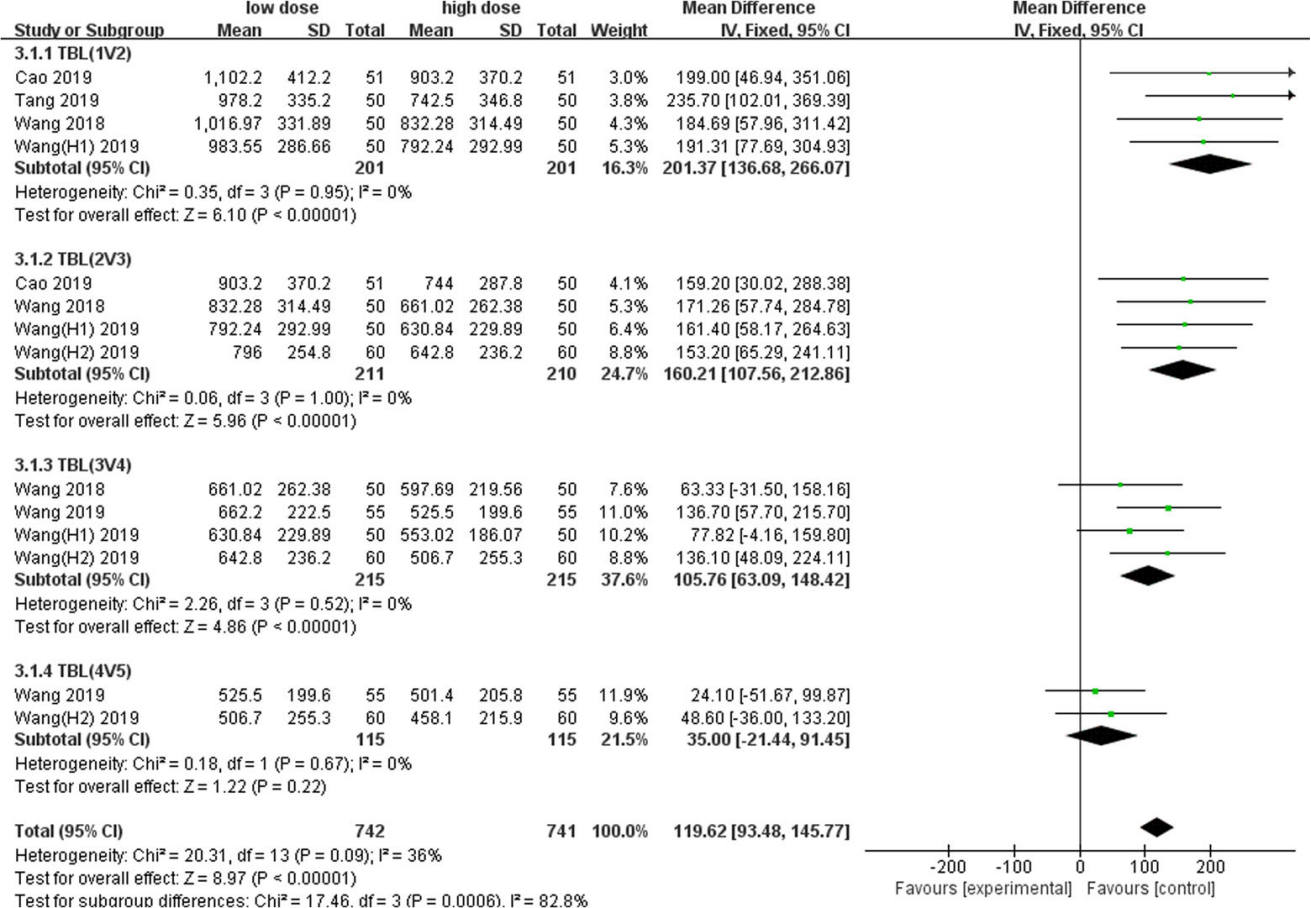
Forest plot showing the weighted mean difference in total blood loss (TBL) between low- and high-dose groups

**Fig. 11:**
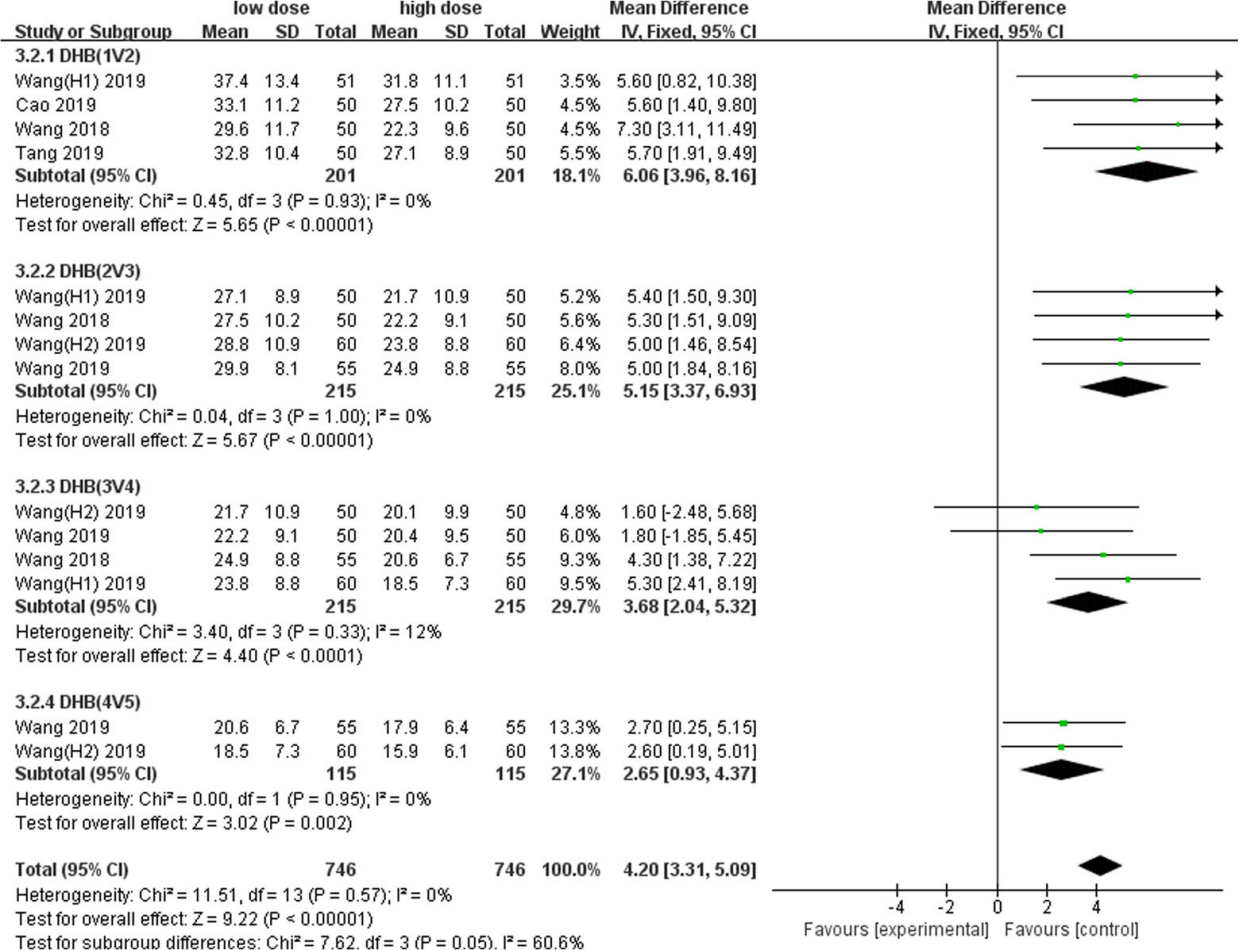
Forest plot showing the weighted mean difference in the decline of hemoglobin (DHB) between low- and high-dose groups

Nine studies involving 1678 patients were included in this meta-analysis. Our results showed that oral TXA could significantly reduce the total blood loss (TLB), intraoperative blood loss (IBL), decline of hemoglobin (DHB), length of hospital stay (LOS), and transfusion rate ([MD = − 496.94, 95%CI (− 611.41 to − 382.47), *P* < 0.001]; [MD = − 118.5, 95% CI (− 166.77 to − 70.24), *P* < 0.001]; [MD = − 13.06, 95% CI (− 17.67 to − 8.45), *P* < 0.001]; [MD = − 0.12, 95% CI (− 0.17 to − 0.06), *P* < 0.001]; [OR = 0.26, 95% CI (0.16 to 0.43), *P* < 0.001]) and did not increase the incidence of DVT and IVT in total joint replacement patients ([OR = 1.05, 95%CI (0.58 to 1.88), *P* = 0.88]; [OR = 1.62, 95% CI (0.52 to 4.99), *P* = 0.40]). Moreover, the effectiveness of oral TXA administration increases as the number of administrations increases. Compared with one administration of oral TXA, two administrations of TXA significantly reduced total blood loss (TBL) and decreased hemoglobin (DHB). In addition, three administrations of oral TXA were more effective than two administrations of oral TXA, and four administrations of TXA were better than three administrations of TXA. Six studies suggested that a greater number of administrations of oral TXA administration were more effective at decreasing the blood loss (Figs. 3, 4, 5, 6, 7, 8, 9, 10 and 11).

## Discussion

Our results showed that compared with placebo, oral TXA could significantly reduce the total blood loss, intraoperative blood loss, decline of hemoglobin, transfusion rate, and length of hospital stay. In addition, oral TXA did not increase the incidence of DVT and IVT for total joint replacement patients. Furthermore, the effectiveness of oral TXA administration increases as the number of administrations increases, and there was no evidence that oral TXA was associated with a high risk of side effects. Thus, we considered oral TXA to be an effective method to reduce blood loss, and a greater number of administrations of oral TXA at different time points during the operation were more beneficial for patients who received total joint arthroplasty.

Perioperative blood loss is a common complication for total joint arthroplasty patients, which might result in swelling and stiffness and prolong the time of rehabilitation [24, 25]. To overcome this problem, various techniques have been used to control blood loss during surgery. Currently, allogeneic blood transfusions are a popular and effective method for improving patients’ postoperative HB levels and are accepted in many major orthopedic surgeries [26–28]. However, transfusion might result in the reaction of the immune system and increase the risk of infection, and several side effects have been reported in previous studies. Thus, there is an urgent need for an effective and safe method to help reduce blood loss during surgery [29, 30].

TXA is an antifibrinolytic drug that has been demonstrated to effectively reduce total blood loss for patients who received major orthopedic surgery in many previous publications [31]. However, the majority of reports have focused on topical and intravenous TXA administration or the combination of the two application methods, and many previous studies have confirmed that there are no significant differences in terms of effectiveness and safety in decreasing blood loss and transfusion rates between the two administration procedures. However, little is known about oral TXA administration in primary total joint replacement, and some researchers believe that multiple administrations or high doses of TXA can maintain the drug concentration and result in better outcomes. To date, however, there is not enough evidence to support these views [32, 33]. Thus, we conducted this research to investigate the relationship between multiple administrations of oral TXA and blood loss in total joint replacement patients. To ensure the accuracy of the results, only RCTs were included in our research. Our results suggested that oral TXA was an effective method to control perioperative blood loss and did not increase the risk of IVT or DVT. In addition, we found that the effectiveness of oral TXA administration increases as the number of administrations increases and that there was an upper limit (five administrations). Furthermore, we found that five administrations lead to better outcomes than four administrations, but there is a lack of enough data for analysis (only two reports); therefore, there is a need for more high-quality RCT studies in the future to support this view.

Although oral TXA was associated with an increased reduction of the blood loss, there is still no consensus on the optimal timing and total dose of oral TXA. In our research, the included studies reported different standard applications of oral TXA. Six studies recommended 2 g TXA as the basic dose, and one recommended 1 g, while two studies adopted 20 mg/kg doses of TXA. In addition, the included studies administered TXA at different time points between pre- and postoperation, and none of the studies reported data beyond 24 h postoperatively. The repeated administrations included two, three, four, and five administrations. Furthermore, there was no standard for the total dose of TXA application because of the different timing and dosing standards applied in the included studies; therefore, we consider that it is difficult to determine the best total dose and time point of oral TXA administration in total joint replacement patients. However, through our gross analysis, the results demonstrated that 2 g TXA as a basic dose was an effective and safe method to reduce blood loss during total joint replacement and that repeated administrations in the first 24 h postoperatively are more effective.

Many previous studies have demonstrated the safety and efficacy of TXA administration through different routes in TKA or THA, but several complications remain, especially the incidence of deep vein thrombosis (DVT) and intramuscular venous thrombosis (IVT) [34]. In addition, some studies considered that intravenous TXA application was associated with cardiovascular disease or renal dysfunction, which have not been recorded in studies of oral TXA [35]. Our results suggested that there were no significant differences in terms of the incidence of DVT and IVT between the placebo and oral TXA treatments (including all more repeat administration groups). Thus, we thought more administrations of oral TXA did not increase the risk of DVT and IVT compared with the placebo group.

However, this research still has some limitations. First, this meta-analysis only included nine studies, and the surgeons all came from China. Most of them were working in the same hospital (West China Hospital), which may have caused bias in the results. Second, the dosing and timing of TXA administration differed across studies, which may have affected the accuracy of the final results. Third, all included studies lacked long-term follow-up, which may have decreased the reliability of the results. Although this meta-analysis contains various limitations, all included studies were high-quality RCT studies, which means that the results are still powerful enough to support our views. Nevertheless, more multicenter, prospective, randomized control trials with large sample sizes are needed in the future.

## Conclusion

Our results suggested that oral TXA could significantly reduce the blood loss, length of hospital stay, and does not increase DVT and IVT incidence for total joint replacement patients; meanwhile, more times of oral TXA administration are more effective.

## Data Availability

All authors consent for availability of data and materials.

## Abbreviations

TXA: Tranexamic acid
TBL: Total blood loss
DHB: Decline of hemoglobin
HS: Hospital stay
IVT: Intramuscular venous thrombosis
DVT: Deep vein thrombosis
TKA/THA: Total knee/hip arthroplasty
TKR/THR: Total knee/hip replacement
RRs: Relative risks
CIs: Confidence intervals
